# Transition of patients with systemic lupus erythematosus from pediatric to adult-oriented rheumatology care

**DOI:** 10.55730/1300-0144.5900

**Published:** 2024-11-05

**Authors:** Seher ŞENER, Gözde Kübra YARDIMCI, Ezgi Deniz BATU, Levent KILIÇ, Ümmüşen Kaya AKÇA, Müşerref Kasap CÜCEOĞLU, Zeynep BALIK, Özge BAŞARAN, Yelda BİLGİNER, Şule APRAŞ BİLGEN, Seza ÖZEN

**Affiliations:** 1 Division of Rheumatology, Department of Pediatrics, Faculty of Medicine, Hacettepe University, Ankara, Turkiye; 2 Division of Rheumatology, Department of Internal Medicine, Faculty of Medicine, Hacettepe University, Ankara, Turkiye

**Keywords:** Adult care, juvenile-onset, pediatric care, systemic lupus erythematosus, transition

## Abstract

**Background/aim:**

The transition from pediatric to adult-oriented care for individuals with juvenile-onset systemic lupus erythematosus (SLE) poses significant challenges. This study aimed to assess the outcomes of transitioning patients with juvenile-onset SLE from pediatric to adult-oriented care.

**Materials and methods:**

Patients with juvenile-onset SLE were included in the study. They were transferred in face-to-face meetings where at least one pediatric rheumatologist and one adult rheumatologist were present (transition time: October–December 2020).

**Results:**

The median (25th–75th percentile) age at diagnosis and the time of the first examination in an adult-oriented rheumatology department of the included 65 SLE patients were 14.3 (10.9–15.1) years and 19.2 (18.5–20.4) years, respectively (female/male ratio: 7.1). There was no difference in clinical findings related to SLE between the last pediatric care visit and the last adult-oriented care visit other than constitutional symptoms being more prevalent in adult-oriented care (p = 0.039). There was a higher rate of low medication adherence in the post- than pretransition period (p = 0.003). The number of patients admitted to the emergency department during follow-up in adult-oriented care was higher (p = 0.009). Additionally, patients were more likely to miss at least one scheduled appointment in the post- than pretransition period (p = 0.002).

**Conclusion:**

We observed that patients with juvenile-onset SLE had more constitutional symptoms, lower medication compliance, higher rates of emergency department visits, and more missed appointments in the posttransition period despite a face-to-face structured transition process. We hope that future studies will offer solutions to the problems in transitional care.

## 1. Introduction

Juvenile-onset systemic lupus erythematosus (SLE) is defined as SLE with onset before the age of 18 years and it accounts for approximately 20% of all SLE cases [1–3]. Juvenile-onset SLE typically occurs during adolescence, a susceptible period of development, with women, some racial/ethnic groups, and individuals of low socioeconomic status being particularly affected [4]. These factors make transitioning SLE patients from pediatric care to adult care challenging.

Patients with juvenile-onset SLE have long durations of overall survival with minimal morbidity thanks to advances in SLE treatment [5]. With studies conducted in recent years, the importance and awareness of the transition from pediatric care to adult-oriented care have increased [6–8]. Despite this, data on long-term outcomes focusing on the impact of care transition in juvenile-onset SLE are sparse [6,8,9]. Patients with juvenile-onset SLE could face barriers to a successful transition similar to those demonstrated for other chronic conditions [7,10]. However, the disease also presents unique challenges due to its complex pathogenesis, multisystem involvement, and onset during adolescence [2,11]. It can be argued that patients with juvenile-onset SLE are among those most likely to struggle with care transitions and are highly likely to suffer significant morbidity and even mortality if their medical care is interrupted.

The transition of patients with SLE from pediatric to adult-oriented rheumatology care is a crucial phase in managing this lifelong autoimmune disease. Successful transition involves early planning, education on self-management, gradual transfer, multidisciplinary collaboration, and ongoing psychosocial support [7,8]. Transition planning should involve specialists including nephrologists, dermatologists, and neurologists to maintain cohesive and comprehensive care [7]. A structured approach helps prevent treatment lapses, improves adherence, and supports better long-term outcomes [6]. After transition, follow-up evaluations assess the patient’s adaptation to adult care, disease management, and treatment adherence [8,10]. These assessments can identify issues early, helping to prevent flare-ups or complications.

A better understanding of the experiences and difficulties faced by patients, especially in adult-oriented care, is only possible by closely monitoring the transition process and its aftermath [6,10,12]. The objective of the present study was to evaluate the processes of transition from pediatric rheumatology care to adult-focused rheumatology care in a cohort of patients with juvenile-onset SLE. The study encompassed the pretransition, transition, and posttransition periods.

## 2. Materials and methods

This was a prospective single-center study. The study received ethical approval from the ethics committee of our center (approval number: GO 21/485). Before participating in the study, all patients and their parents provided written informed consent.

### 2.1. Patients

Patients with juvenile-onset SLE were included in this study. Although there is no clearly defined age for transition from pediatric to adult-oriented care, all of our patients were transferred after 18 years of age. The time of transfer was decided for each patient by the primary treating pediatric rheumatologist. Study inclusion criteria were as follows: 1) SLE diagnosis according to the Systemic Lupus International Collaborating Clinics (SLICC) criteria [13], 2) age at diagnosis of <18 years and current age of ≥18 years, 3) follow-up by a pediatric rheumatologist for at least 1 year before the transition to adult care, and 4) having ≥1 visit within a year after their first posttransition visit in adult-oriented rheumatology care.

### 2.2. Transition process

The patient’s medical history was relayed in face-to-face meetings with at least one pediatric rheumatologist (one of whom was the primary doctor of the patient) and an adult rheumatologist in the same center. In addition, the first physical examination of the patient after the transition was performed by these two specialists together. This transition process encompassed the period between October and December 2020. In adult-oriented care, patients were followed for at least 2 years by the adult rheumatologist.

To ensure continuity and quality of care, appropriate precautions were applied while transitioning SLE patients from pediatric to adult care face-to-face during the pandemic. During this process, careful planning and safety measures were implemented to minimize the risk of coronavirus disease 2019 (COVID-19) exposure. Before each visit, all patients were asked about COVID-19 symptoms and their temperatures were taken. Patients in the risk group were excluded from the study and their transitions were postponed. Both patients and healthcare providers wore protective masks throughout the visits. In some cases, gloves and face shields were also used depending on the nature of the examination. Patients and healthcare providers adhered to hand hygiene rules during the examinations. Patients were accompanied by only one caregiver or family member during the visit to minimize exposure. Appointments were staggered to prevent crowding in waiting rooms. For high-risk patients, appointments were scheduled for specific hours or days when fewer patients were present. Patients were escorted directly to an examination room upon arrival, ensuring that they spent as little time as possible in common areas.

Sex, age at symptom onset and diagnosis, age at the first and last visit in adult-oriented rheumatology care, level of education, socioeconomic status, availability of health insurance, and follow-up periods in pediatric and adult-oriented rheumatology care were recorded and evaluated for all patients. Average annual household income in Türkiye was determined according to 2021 census data. SLE-related clinical and laboratory findings, treatments, drug compliance, visits to the emergency department, missed appointments, and remission status of the patients were evaluated and compared at the last pediatric care visit and the last adult-oriented rheumatology visit. In addition, the Systemic Lupus Erythematosus Disease Activity Index (SLEDAI) [14] and the SLICC Damage Index [13] were calculated for both visits. The Morisky Medication Adherence Scale-4 (MMAS-4) was used to evaluate patients’ medication compliance before and after the transition [15]. The MMAS-4 utilizes a scoring system whereby each item is assigned a value of 1 (indicating “no”) or 0 (indicating “yes”). The total score, ranging from 0 to 4, is determined by summing the scores for each item. According to the scoring system, a score of 0 points or 1 point reflects low adherence, a score of 2 or 3 points signifies medium adherence, and a score of 4 points signifies high adherence. Remission was defined as both clinical and serological inactive disease (SLEDAI of <4) for ≥12 months with or without immunosuppressive therapy.

### 2.3. Statistical analysis

Statistical analyses were conducted using IBM SPSS Statistics 25.0 (IBM Corp., Armonk, NY, USA). Numerical variable distributions were assessed for their compliance with normality using visual methods (histograms, probability plots) and the analytical Shapiro–Wilk test. The results of descriptive analyses were presented as mean ± standard deviation values or median (25th–75th percentile) values for continuous variables and as numbers and percentages for categorical variables. Differences in proportions between groups were assessed using the chi-square test or Fisher exact test where appropriate. The Mann–Whitney U test was employed to compare nonnormally distributed continuous data across different groups. Values of p < 0.05 were accepted as statistically significant.

## 3. Results

### 3.1. Patients’ general characteristics

Sixty-five patients with juvenile-onset SLE (female/male: 7.1) were transferred from pediatric to adult-oriented rheumatology care in our center. At the first posttransfer visit to the adult-oriented rheumatology care, the pediatric rheumatologist who had previously followed the patients primarily was also involved. The patients’ median ages at symptom onset and diagnosis were 13.2 (10.5–14.9) and 14.3 (10.9–15.1) years, respectively (Table 1). The majority of patients had a university education (n = 42, 64.6%), health insurance (n = 51, 78.5%), and moderate annual household income (n = 49, 75.4%) at the time of transition. The patients’ median age at the first and last adult-oriented rheumatology examinations were 19.2 (18.5–20.4) and 21.8 (20.6–24.3) years, respectively. After the transfer, the patients were followed in the adult-oriented rheumatology department for a mean of 2.5 ± 0.8 years.

### 3.2. Differences between pediatric and adult-oriented care visit

There was no difference in SLE-related clinical and laboratory features between the last pediatric care visit and the last adult-oriented care visit other than constitutional symptoms, which were more frequently reported during the last visit to adult-oriented rheumatology care (p = 0.039) (Table 2). The SLEDAI and SLICC scores also did not differ significantly from the beginning to the end of the posttransition period (both p = 1.000) (Table 3).

Medication nonadherence was observed at a higher rate in the posttransition period (p = 0.003) (Table 3). More patients had low medication adherence at the last adult-oriented care visit than at the last pediatric care visit (p = 0.041). The most frequently discontinued drug was hydroxychloroquine (p = 0.001).

The number of patients visiting the emergency department was higher in adult-oriented rheumatology care (n = 16 [24.6%] versus n = 5 [7.7%], p = 0.009). Most of these visits were due to SLE-related problems (75%) and patients stated that they had difficulty in making an appointment at the adult-oriented rheumatology outpatient clinic.

Nearly one-third of the patients missed at least one scheduled appointment in adult-oriented rheumatology care in the posttransfer period, and this rate was higher than the rate observed in pediatric care (n = 19 [29.2%] versus n = 5 [7.7%], p = 0.002).

Before the transition, only five patients were not in complete remission. Two of these five patients had isolated serological activation, one had active rash (urticaria), one had intermittent arthritis, and one had hematological involvement (leukopenia). After the transition, ten patients had active disease. Three of these patients had isolated serological activation, two had hematological involvement (leukopenia and thrombocytopenia), two had mucocutaneous involvement, one had arthritis, one had serositis, and one had active renal involvement. No significant difference was detected between the pediatric and adult-oriented care periods in terms of recent remission status (p = 0.062).

## 4. Discussion

In this study, we evaluated the long-term outcomes of transition from pediatric to adult-oriented rheumatology care in a juvenile-onset SLE cohort. No significant difference was detected between SLE-related clinical findings (except for constitutional symptoms), laboratory results, and disease activity scores after the patients were transferred from pediatric to adult-oriented care. In contrast, low medication compliance, more emergency department visits, and at least one missed appointment were more frequently observed in the adult-oriented care period.

In this study, no deterioration in SLE-related clinical findings other than constitutional symptoms was observed between the pediatric care and adult-oriented care follow-up periods of juvenile-onset SLE patients. Although there are a limited number of studies in the literature evaluating the care transitions of juvenile-onset SLE patients [6,8,16], noteworthy results were obtained in terms of worsening clinical findings after the posttransition period, particularly in the study conducted by Felsenstein et al. [8]. They evaluated the transition from pediatric to adult-oriented care of 41 juvenile-onset SLE patients with a mean age of 24 ± 4.2 years and a mean follow-up duration of 5 ± 3.7 years (female/male ratio: 9.2). More than half of the patients (n = 22, 53.6%) experienced poor symptom control (p = 0.030) and more symptoms associated with the involvement of multiple organ systems (p = 0.050) at follow-up. After the transition, 17 patients (41.5%) exhibited newly developed renal involvement. Approximately one-third (n = 15, 36.6%) reported new or ongoing neuropsychiatric symptoms [8]. However, in another study evaluating the transitions of 50 juvenile-onset SLE patients, although one-quarter of the patients had active renal disease during the transition period, only two patients had active renal disease at the end of the posttransition period [6].

No difference was detected between the SLEDAI and SLICC scores of our juvenile-onset SLE patients before and after the transition in the present study. Consistent with our findings, Hersh et al. [17] found that SLEDAI scores were stable before and after transition in 16 patients with juvenile-onset SLE. On the other hand, although SLEDAI scores were found to be moderate and stable with both types of care, SLICC scores were found to be significantly higher in the posttransition period of juvenile-onset SLE patients in another study [6].

The most important findings of our study were low medication compliance in the posttransfer period and an increase in the frequency of emergency department visits and missed appointments. Most of the emergency department visits were due to SLE-related problems. These differences may be due to difficulty in making appointments with adult care, inability to communicate directly with the adult physician, or patients not always being evaluated by the same rheumatologist during visits in adult care.

In a study conducted by Son et al. [6], 50 juvenile-onset SLE patients who were transferred from pediatric to adult-oriented care were evaluated (female/male ratio: 9.0). The mean ages of the patients at the time of symptom onset and diagnosis were 14.1 (range: 6–18) years and 14.5 (range: 6–18) years, respectively. Approximately two-thirds of patients (n = 32, 64%) had been followed for at least 2 years. Medication nonadherence was more frequent in the post- than pretransition period (n = 6 [12%] vs. n = 12 [24%], p = 0.010). Emergency department visits in the posttransition period were made by 15 patients (30%) in that study, with the majority of admissions (77%) resulting from SLE or treatment-related complications, consistent with our study. However, unlike our study, no difference was detected in the number of emergency department admissions before and after the care transition. Additionally, nearly half of the patients (n = 23, 46%) in that study had missed at least one scheduled appointment during the posttransition period [6]. In Poisson regression analysis, education below high school level, noncompliance with medication, and white race were found to be significantly associated with missed appointments.

The transition from pediatric to adult-oriented care has been shown to negatively impact outcomes in patients with a variety of chronic diseases, including SLE [8,18]. Our study results were consistent with previous observations in this regard. Successful transition from pediatric to adult-oriented care for all youth with chronic conditions depends on the individual’s self-management capacity and the medical center’s ability to provide patient-centered care [19,20]. In this context, the severity of chronic diseases, the responsibilities of adulthood, and complex medical systems must be considered [21]. The methods that will successfully guide these patients through the transition process must be tailored to each case and can be implemented by the patient and the care center.

The major limitation of this study is that the last follow-up visit of some patients was performed by an adult rheumatologist other than the primary physician. In adult-oriented care, patients were followed for at least 2 years by the primary adult rheumatologist. However, the primary adult rheumatologist of the patients had recently been transferred to another center. Therefore, the final evaluation of some patients was performed by a different adult rheumatologist. Another important limitation was that the increase in emergency department visits was due to difficulties in making appointments in the adult-oriented rheumatology department. There was no such difficulty in pediatric rheumatology care. All SLE patients were transferred at ≥18 years old. However, this age limit is not set in stone and may vary among publications in the literature. In addition, since our study population may not be representative of the general population, it is difficult to generalize the diagnostic findings. Finally, the difference in follow-up durations between pediatric and adult-oriented rheumatology departments might have affected the results.

In conclusion, according to our long-term results regarding care transition in a cohort of juvenile-onset SLE patients, an increase in constitutional symptoms, lower medication compliance, more frequent emergency department visits, and a higher frequency of missed appointments were observed in the posttransition period. These results show that there were some problems in the transition process. We hope that future studies conducted with larger cohorts and reflecting longer-term results will address the problems, incompatibilities, and complexities in care.

## Figures and Tables

**Table 1:** Demographic and social characteristics of patients with juvenile-onset systemic lupus erythematosus.

n (%) or median (25th–75th percentile) or mean ± SD	SLE patients (n = 65)

Sex, female	57 (87.7)

Age at symptom onset, years	13.2 (10.5–14.9)

Age at diagnosis, years	14.3 (10.9–15.1)

Educational level of the patient at the time of transfer	
<High school	3 (4.6)
High school	19 (29.3)
University	42 (64.6)

Classification according to mean annual household income	
Low	9 (13.8)
Moderate	49 (75.4)
High	7 (10.8)

Number of patients who had health insurance	51 (78.5)

Age at first visit to adult-oriented rheumatology care, years	19.2 (18.5–20.4)

Age at last visit to adult-oriented rheumatology care, years	21.8 (20.6–24.3)

Days from last pediatric to first adult-oriented care visit	19 (14–27)

Duration of follow-up in pediatric rheumatology care, years	4.8 (2.7–6.5)

Duration of follow-up in adult-oriented rheumatology care, years	2.5 ± 0.8

SD: Standard deviation; SLE: Systemic lupus erythematosus.

**Table 2:** Differences of systemic lupus erythematosus-related clinical and laboratory findings between the patients’ last pediatric care visit and last adult-oriented care visit.

n (%) or median (25th–75th percentiles)	At last pediatric care visit (n = 65)	At last adult-oriented care visit (n = 65)	p

SLE-related findings			
Constitutional symptoms	8 (12.4)	17 (26.2)	0.039
Mucocutaneous findings	6 (9.3)	7 (10.8)	0.770
Arthritis	3 (4.6)	2 (3.1)	1.000
Serositis	0	1 (1.5)	-
Hematologic involvement^a^	11 (16.9)	9 (13.8)	0.627
Renal involvement^a^	7 (10.8)	8 (12.3)	0.784
Neuropsychiatric involvement^a^	2 (3.1)	2 (3.1)	1.000

Laboratory findings			
Hemolytic anemia	2 (3.1)	1 (1.5)	1.000
Leukopenia (<4000/mm^3^)	6 (9.3)	7 (10.8)	0.770
Thrombocytopenia (<100,000/mm^3^)	3 (4.6)	2 (3.1)	1.000
Hypocomplementemia	9 (13.8)	11 (16.9)	0.627
ANA positivity (>1/100)	61 (93.8)	59 (90.7)	0.510
Anti-dsDNA positivity	7 (10.8)	9 (13.8)	0.593
Positive direct Coombs test	3 (4.6)	3 (4.6)	1.000
Anti-Sm positivity	5 (7.7)	6 (9.3)	0.753
Lupus anticoagulant	11 (16.9)	12 (18.5)	0.818
Anti-cardiolipin IgM/IgG positivity	5 (7.7)	4 (6.2)	1.000
Anti-β2-glycoprotein IgM/IgG positivity	4 (6.2)	4 (6.2)	1.000

ANA: Antinuclear antibody; anti-dsDNA: Anti-double-stranded DNA; Anti-Sm: Anti-Smith; Ig: Immunoglobulin; SLE: Systemic lupus erythematosus.

a Currently continuous or new findings.

**Table 3:** Differences of disease activity and damage index, drug compliance, treatments, and outcomes between patients’ last pediatric care visit and last adult-oriented care visit.

SLEDAI	1 (0–3)	1 (0–4)	1.000

SLICC	0 (0–1)	0 (0–1)	1.000

Treatments			
Hydroxychloroquine	65 (100)	54 (83.1)	0.001
Glucocorticoid	27 (41.5)	33 (50.8)	0.291
≤10 mg/day	24 (36.9)	28 (43.1)	0.474
>10 mg/day	3 (4.6)	5 (7.7)	0.718
Mycophenolate mofetil	7 (10.8)	5 (7.7)	0.545
Azathioprine	2 (3.1)	2 (3.1)	1.000
IVIG	2 (3.1)	1 (1.5)	1.000

Drug compliance (MMAS-4)			
High	56 (86.2)	41 (63.1)	0.003
Medium	6 (9.2)	14 (21.5)	0.052
Low	3 (4.6)	10 (15.4)	0.041

Remission	60 (92.3)	55 (84.6)	0.170

IVIG: Intravenous immunoglobulin; MMAS-4: Morisky Medication Adherence Scale-4; SLEDAI: Systemic Lupus Erythematosus Disease Activity Index; SLICC: Systemic Lupus International Collaborating Clinics.
